# From 3D to 2D and back again

**DOI:** 10.1515/nanoph-2022-0512

**Published:** 2023-01-04

**Authors:** Niyazi Ulas Dinc, Amirhossein Saba, Jorge Madrid-Wolff, Carlo Gigli, Antoine Boniface, Christophe Moser, Demetri Psaltis

**Affiliations:** Optics Laboratory, École polytechnique fédérale de Lausanne, Lausanne, Switzerland; Laboratory of Applied Photonics Devices, École polytechnique fédérale de Lausanne, Lausanne, Switzerland

**Keywords:** 3D optical memory, additive manufacturing, inverse design, optical tomography, photonic circuit design

## Abstract

The prospect of massive parallelism of optics enabling fast and low energy cost operations is attracting interest for novel photonic circuits where 3-dimensional (3D) implementations have a high potential for scalability. Since the technology for data input–output channels is 2-dimensional (2D), there is an unavoidable need to take 2D-nD transformations into account. Similarly, the 3D-2D and its reverse transformations are also tackled in a variety of fields such as optical tomography, additive manufacturing, and 3D optical memories. Here, we review how these 3D-2D transformations are tackled using iterative techniques and neural networks. This high-level comparison across different, yet related fields could yield a useful perspective for 3D optical design.

## 1 Introduction

Optical information processing is an attractive topic for scientists and researchers due to the potential fast and energy-efficient performance guaranteed by the intrinsic physical properties of optics [1]. With the advancements in micro/nano fabrication, nowadays implementing photonic circuitry is becoming more and more a reality. However, the field still stays infant and requires breakthroughs. Along with integrated solutions [2–5], one of the promising ways of taking advantage from the parallelism of optics is using 3-Dimensional (3D) implementations, which enable the scalability of the systems [6–9]. Nonetheless, the data injection and read-out systems, such as spatial light modulators and detectors are at best 2-dimensional (2D); hence, it subsist an imperative necessity for transformations between 3D and 2D for both illuminating and collecting information with light. This is also the case for human vision. We live in a 3D world but we rely on a set of 2D sensors (the retinas in our eyes). Therefore, the human neural vision system adapted to perform an incredible job; from only a set of two 2D projections at a slightly different angle, our brain can reconstruct the 3D scene. Following the machinery of evolution, one would expect artificial neural networks to have a similar role in carrying out these transformations. It is therefore worthy to take a step back to enumerate and understand the problems related to 3D-2D transformations.

In this paper, we first review optical tomography, which is one of the most prominent methods for 3D imaging dealing with the reconstruction of volumetric objects from 2D recordings. The inversion of the scattering problem, at the core of this technique, is severely hampered by the limited number of available projections, at the origin of the so-called “missing cone”, which makes the transformation back to 3D an ill-posed problem, and multiple scattering occurring within the object. Neural networks are frequently employed to unscramble and fill in the missing information using data-driven (statistical) and physics-based approaches with different techniques presented in Section 2. Then we will review volumetric additive manufacturing, where the problem is reversed by using a tomographic-based method to fabricate 3D objects in a fast and effective manner. This part reports on an example of going from 3D (known target object) to 2D (unknown corresponding projections) and back again to 3D (fabricated physical object). In the same manner, as the printing process leverages the transfer of information from 2D projections to shape a volumetric object, one can pattern the matter in 3D to store 2D data, such as collections of pages, matrices, images, and so on. In Section 4, we review 3D optical memories as another example where the 3D information is retrieved from 2D measurements. In this case, we have 2D input–output planes and a 3D medium that specifies the different mappings between the input–output planes.

Finally, in Section 5, we outline the recent approaches using neural networks and other iterative optimization schemes for designing 3D optical circuitry, which unavoidably performs 2D-to-2D mappings using 3D features of optics. We believe that understanding how other fields deal with the fundamental challenges arising from 3D-2D transformations and how neural networks are used in these fields could provide a valuable perspective for optical/photonic circuit design and fabrication. Photonics, in this regard, would be most beneficial for neural network architectures particularly when massive parallelism is required, which establishes interesting positive feedback between two fields.

## 2 Optical tomography

**Figure 1:**
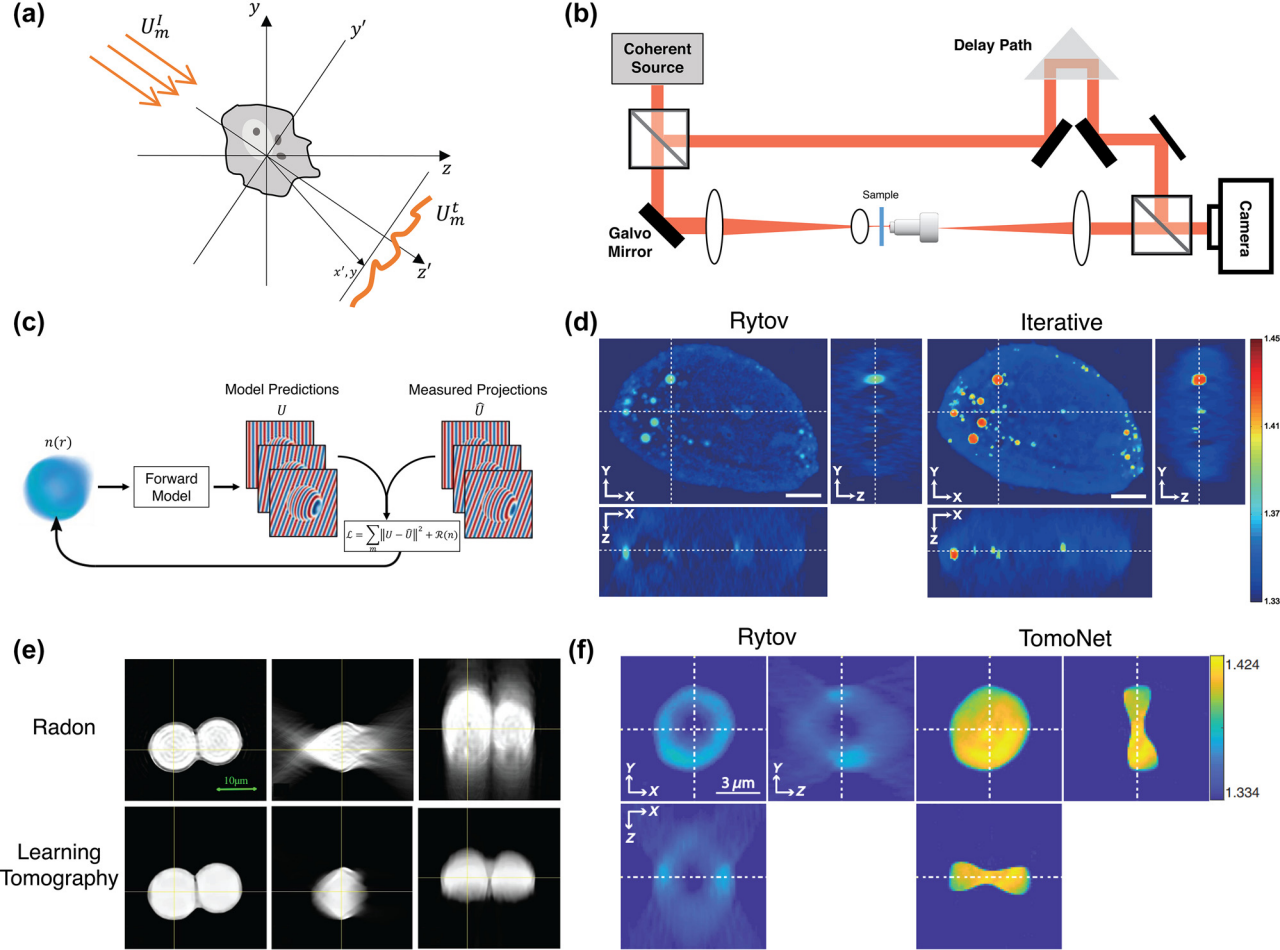
Optical tomography. (a) An overview of the optical tomography problem. A 3D object is illuminated with different plane waves, and 2D quantitative phase projections are measured for each illumination angle. (b) A standard off-axis holography setup for refractive index tomography. The illumination angle can be controlled using a pair of galvo mirrors. (c) Iterative optical diffraction tomography (ODT): A forward model (such as single-scattering [23] or beam propagation method [24] computes the 2D projections for each illumination angle. By comparing this field to the measurements, a loss function is calculated, which is minimized by improving the reconstruction of the 3D refractive index iteratively. (d) Comparison of ODT reconstruction results for a hepatocyte cell using the Rytov approximation and iterative ODT with edge-preserving regularization (Adapted from [23] Copyright OPTICA). The scale bar is 5 µm. (e) Tomographic results of two 10 µm polystyrene beads immersed in oil with *n*_0_ = 1.516 based on inverse Radon transform and learning tomography (Adapted from [24], Copyright OPTICA). (f) 3D reconstruction of a red blood cell using TomoNet. (Adapted from [28] Copyright SPIE). Figures (e) and (f) show that learning tomography and TomoNet solve underestimation and elongation of the reconstructions.

Optical tomography is an example of an imaging method that reconstructs the 3D refractive index distribution of the sample using multiple 2D projections. Here, 2D projections correspond to quantitatively measured scattered fields acquired by illuminating the sample from different angles as shown in Figure 1(a). The sample, with 3D refractive index distribution *n*(*r*), is illuminated with a set of plane-waves $U_{m}^{I}={\text{e}}^{j\vec{k_{m}}.\vec{r}}$ , and the complex fields, $U_{m}^{t}$ , are measured for each projection, *m*. The refractive index of the sample is locally correlated with the mass density, which makes its 3D reconstruction interesting for a variety of biological applications [10, 11].

Conventionally, the 2D projections are measured in an off-axis holography configuration to capture both amplitude and phase information of the scattered field. A standard optical tomography setup is presented in Figure 1(b) where a coherent and collimated visible source is divided into a signal and reference beams with a beam-splitter. The angle of the signal beam is controlled using a pair of galvo-mirrors, and a 4F system is used to magnify the illumination angle. The illuminated sample is then imaged onto a camera through another 4F system consisting of a microscope objective and a tube lens. The off-axis reference beam and the signal beam are recombined to form the hologram on the detector plane. We can process the holograms in the Fourier domain to retrieve the phase and amplitude of the complex projections.

The optical scattering can be described by the Helmholtz equation in an inhomogeneous medium [12]:

(1) $$\nabla^{2}U^{\text{s}}\left(r\right)+{\left(k_{0}n_{0}\right)}^{2}U^{\text{s}}\left(r\right)=-V\left(r\right)U\left(r\right)$$

where *U*^s^(*r*) is the scattered field, *k*_0_ is the wave number in free space, *n*_0_ is the refractive index of the background medium, $U\left(r\right)=U^{\text{s}}\left(r\right)+U^{I}\left(r\right)$ is the total field, and $V\left(r\right)={\left(k_{0}n_{0}\right)}^{2}\left(n^{2}\left(r\right)/n_{0}^{2}-1\right)$ is the scattering potential of the sample. The integral solution of *U*^s^(*r*) based on Eq. (1) is nonlinear with respect to the scattering potential, and as a result, the optical scattering problem cannot be directly inverted to achieve the 3D scattering potential. Additionally, due to the finite number of projections, and limited-numerical aperture (NA) of the imaging system, there is missing information that makes the inverse problem more difficult. In the following, we summarize ray-optics-based and single-scattering approximations that linearize the 3D scattering potential reconstruction problem, and then we review optimization and machine learning techniques for addressing missing information and multiple-scattering problems.

### 2.1 Optical tomography based on direct inversion

Charrière et al. [13], and Choi, et al. [14] reported the first experimental implementation of tomographic refractive index reconstruction for biological cells. Even though optical diffraction tomography was theoretically proposed and elaborated much earlier, the refractive index reconstruction method in [13, 14] is based on the ray-optics approximation. If we assume weakly diffractive objects, the phase of a 2D projection in $U\left(r^{\prime }\right)=U^{I}\left(r^{\prime }\right){\text{e}}^{j\varphi \left(r^{\prime }\right)}$ will be proportional to the integration of *δn*(*r*), the refractive index contrast of the sample with respect to the background medium, along the optical axis [13]:

(2) $$\varphi \left(x^{\prime },y^{\prime },z^{\prime }=z_{0}\right)=\overset{z_{0}}{\int }k_{0}\delta n\left(r^{\prime }\right)dz^{\prime }$$

Equation (2) is the line integral of the refractive index contrast along the projection direction, which is known as the Radon transform of *δn* [15]. This representation clarifies the similarity to computed X-ray tomography for 3D reconstruction of the absorption using 2D intensity measurements. Having the 2D phase profiles for different illumination angles, an inverse Radon algorithm based on filtered back projection can be used to reconstruct the 3D tomograms of refractive index contrast, *δn*(*r*).

For samples with features comparable to the wavelength, the diffraction of light cannot be neglected. Emil Wolf proposed optical diffraction tomography (ODT) [12] in 1969 using the Born approximation to linearize the integral solution of Eq. (1). Wolf showed that using the Born approximation, the 3D Fourier transform of the scattering potential can be related to the 2D Fourier transform of each projection according to the incident wave-vector,

(3) $$F_{2D}\left\{U_{m}^{\text{s}}\right\}\left(k_{x},k_{y}\right)=\frac{2\pi j}{k_{z}}\tilde{V}\left(k_{x}-k_{x}^{\text{in}},k_{y}-k_{y}^{\text{in}},k_{z}-k_{z}^{\text{in}}\right)$$

where $U_{m}^{\text{s}}$ is the scattered field for projection *m*, *k*_*x*_, and *k*_*y*_ are the spatial frequencies, $k_{x}^{\text{in}}$ , $k_{x}^{\text{in}}$ , and $k_{x}^{\text{in}}$ are the wave vectors of the illumination beam and $k_{z}=\sqrt{k^{2}-k_{x}^{2}-k_{y}^{2}}$ .

We can use Eq. (3) to fill the 3D Fourier domain of the scattering potential. Devaney proposed using the Rytov approximation for ODT [16] by using $U^{\text{I}}\left(r\right)\log\left\{U\left(r\right)/U^{\text{I}}\left(r\right)\right\}$ instead of *U*^s^(*r*) on the left side of Eq. (3) which can be justified with the first-order Taylor expansion. Sung et al. [17] presented the first experimental results on diffraction tomography using the Rytov approximation. Later, many groups thoroughly studied different aspects of ODT such as illumination beam rotation [18], sample rotation [19], temporally incoherent ODT [20], wavelength scanning [21], and polarization-sensitive ODT [22]. In Figure 1(d), a 3D refractive index reconstruction of hepatocyte cells is shown using Wolf’s method with the Rytov approximation [23]. The ill-posed nature of the direct inversion of the scattering problem causes missing frequencies in the Fourier domain of the reconstructed scattering potential. The missing spatial frequencies make the 3D refractive index reconstruction underestimated and elongated along the optical axis. To solve this issue, iterative methods for optical tomography have been investigated.

### 2.2 Machine learning and iterative methods for optical tomography

To consider a more accurate forward scattering model rather than Born or Rytov approximations, and solve the missing frequencies problem, several iterative optimization schemes have been proposed for optical tomography. The main idea of iterative tomography, shown in Figure 1(c) is finding the 3D refractive index distribution by minimization of a loss function, which includes the difference between the field calculated by a forward model and the measured projections, plus a regularization term based on some prior information about the sample,

(4) (4) $$L=\sum_{m}\|U_{m}^{\text{model}}\left(V\right)-{\hat{U}}_{m}^{\text{projection}}{\|}^{2}+R_{\text{prior}}\left(V\right)$$

where $U_{m}^{\text{model}}\left(V\right)$ is the 2D projection calculated for the estimated scattering potential in that iteration using a forward model, ${\hat{U}}_{m}^{\text{projection}}$ is the measured projection, and $R_{\text{prior}}\left(V\right)$ is a regularization term based on prior knowledge of the 3D scattering potential. The iterative optimization for the reconstruction of the refractive index has been presented for ray-optics tomography [14]. For ODT, different regularizers are compared in [23] using a single-scattering forward model. Their results in Figure 1(d) show significant improvement in the underestimation and elongation of the sample, using an edge-preserving regularization term.

A more accurate forward model for the calculation of $U_{m}^{\text{model}}$ is used in [24] based on the beam propagation method. This approach, known as learning tomography, accounts for the multiple scattering and provides a decent 3D reconstruction of the refractive index, as shown in Figure 1(e). This idea was further investigated to achieve 3D reconstructions using a few projections [25], or intensity measurements [26]. Additionally, Tian and Waller demonstrated that LED illumination could be used for tomographic reconstruction with a multi-slice forward model to overcome laser fluctuations and speckle artifacts [27].

Recently, several groups studied machine-learning techniques for ODT. Lim et al. [28] presented a deep neural network, TomoNet, which maps the Rytov-based low axial resolution 3D tomograms to the improved 3D refractive index tomograms. They have generated a dataset of red blood cell phantoms with different sizes, refractive indices, and orientations. Then, they calculated synthetic projections for 40 illumination angles for each phantom by discrete-dipole approximation and calculated Rytov-based reconstruction using these synthetic projections. In such a manner, a dataset of red blood cells with their corresponding Rytov reconstructions can be achieved to train a deep neural network with a U-Net structure. This network, which is trained on synthetic data, can provide 3D tomograms with a reconstruction error two orders of magnitude smaller than Rytov, and it can be also used for experimental projections. In Figure 1(f), the 3D reconstruction of the refractive index of a mouse red blood cell is shown using TomoNet in comparison with the Rytov approximation. Recently, SILACT, a machine learning technique for the 3D reconstruction of the refractive index was presented [29], which is based on a deep neural network that converts a single frame hologram with angle-multiplexing illumination to the 3D refractive index tomogram. In this method, a dataset of input/output pairs is generated as follows: each sample is illuminated with a single frame of angle-multiplexed illumination with four angles, and a raw hologram is measured using off-axis holography. Raw holograms are considered as the input of the network. Then, the sample is illuminated with 49 projections, each from a single angle, and a 3D reconstruction of the sample is calculated using learning tomography based on these projections. This 3D reconstruction is considered as the output of the network. The deep neural network is trained on these input/output pairs. Using the trained deep neural network, a 3D reconstruction of the sample can be achieved with an angle-multiplexed single hologram. Another deep learning method for 3D tomography was recently investigated using a physics-informed neural network, MaxwellNet, as the forward model in Eq. (4) [30]. MaxwellNet minimizes a physics-informed loss function (such as Maxwell equations) and it was originally proposed for an inverse design problem [31]. In contrast to the conventional data-driven neural networks that require a huge dataset, MaxwellNet exploits physical laws to suggest a fast solution to the forward and inverse scattering problems.

Iterative solutions of ODT [23], [24], [25], [26, 30] provide better 3D reconstructions at the cost of computation time. Direct ODT approaches such as Wolf’s method [12] are relatively fast since they require a few operations such as multiplication and fast Fourier transform (FFT) per projection or an additional phase-unwrapping step for each projection in the case of Rytov approximation. However, reconstructing the 3D refractive index tomogram with an iterative optimization method requires performing the forward model for all the projections in each iteration. As a result, depending on the complexity of the forward model and the number of iterations, iterative optical tomography methods are time-consuming. Ref [25] compares Beam Propagation Method (BPM) and Split-Step Non-Paraxial (SSNP) method as forward models for the iterative reconstruction where one iteration (running on a graphics card) takes approximately 3–13 s for BPM depending on the computation volume and 50% more time required for the SSNP version. Note that the required iterations are in the order of a few hundred. More sophisticated forward models such as Lippmann–Schwinger [32, 33], are also used to show high-fidelity reconstructions when the complexity of the data is high at the expense of more computational power. Hence, the time per iteration may differ by an order of magnitude when such models are employed. Moreover, the applied regularization method is also an important factor in the computation time per iteration, which can make a difference by an order of magnitude as shown in Ref. [23]. On the other hand, deep neural networks such as TomoNet [28] and SILACT [29] present tomographic 3D reconstructions of a specific class of samples with a fast inference time, which goes below a second for the whole process of 3D reconstruction of a sample.

Iterative approaches using prior knowledge, accurate forward models such as BPM, and statistical information accomplished by machine learning frameworks can help to achieve a better 3D refractive index reconstruction in the ill-posed optical tomography problem.

## 3 Volumetric printing

3D printing can be thought of as the inverse of tomography in that we know the 3D object and we look for the 2D illuminating patterns that will yield the desired 3D construct. The simplest illumination pattern is a focused spot. Then by scanning this focus spot inside a photo-curable resin, one can fabricate point-by-point (which is a relatively slow process) a 3D structure [34]. A way to speed up the process is to cure the resin layer-by-layer with a series of 2D patterns at different depth [35]. A few years back, a promising and even much faster approach has been developed to 3D print centimeter-scale objects into high-viscosity fluids or even solids in a few tens of seconds with high resolution (<100 µm). The idea, taken up and elaborated simultaneously in two laboratories [36, 37] consists of irradiating the resin with 2D light patterns from multiple angles. The light exposure produces a volumetric dose of energy sufficient to solidify, all at once, the material in the desired geometry, without following a sequential fabrication process as layer-by-layer printers do. Hence, we refer to this method as volumetric additive manufacturing (VAM).

One challenging task is to determine the required light patterns from the desired light dose distribution. An interesting aspect of this two-dimensional inverse problem is its close relationship to computed tomography (CT) which aims at reconstructing a three-dimensional image from its projections as explained in the previous section. Under some simplifying assumptions, 3D imaging and 3D printing are very similar; the problem they represent is simply reversed. It results that we can successfully apply the same 2D-3D transform and use analogous algorithms.

When printing in transparent resins, it was shown that the Radon transform, as used for 3D image reconstructions, can provide a set of 2D patterns to get high-quality 3D prints. The patterns computation workflow as described in [36, 37] consists of, first, converting the target 3D model into a three-dimensional binary matrix of voxels, where the entries “1” indicate the presence of matter and “0” its absence at each particular location in space. Then, for each 2D section of this matrix, projections (also known as “sinogram”) are calculated over multiple angles from the Radon transform using a filtered back-projection algorithm [15]. Additional processing is performed to ensure correct sampling of the projection space and the absence of negative values that cannot be generated with light. It was also proposed to optimize the obtained sinogram to minimize the loss between the target dose and the one obtained from the projections [38, 39].

However, this model, based on the Radon transform, assumes that light patterns propagate straight without being attenuated or distorted inside the photoresist. This would be the case when printing in perfectly homogeneous and fully transparent resins, which does not happen in reality. In essence, the photoinitiator that triggers the chain polymerization absorbs light, leading to an exponential decrease in its intensity with depth. More dramatically, light may also be scattered by the resin because of refractive index inhomogeneities, which is the case for all non-transparent materials. Scattering can strongly affect the spatial propagation of the beam; light deviates from its initial direction which tends to blur the projected patterns and prevents the printing of high-resolution features. Novel methods have recently been proposed to maintain a relatively high resolution for printing while increasing the turbidity/absorptivity of the resin [40, 41]. Based on the inverse Radon transform, a physics-informed forward model is built including resin’s specifications such as the amount of scattering or the degree of absorption and the positivity constraint.

Another way to improve print fidelity is to get additional information during the print. Different imaging systems using bright field [36] or dark field [42] illuminations were proposed to reconstruct the printed object with the standard tomographic algorithm. Such information allows us to stop the print at the right time to avoid over-polymerization of the part but can also be used as feedback to improve the light patterns to be sent for the next prints. In this case, one would adjust the amount of light with respect to polymerized/unpolymerized parts. In the same vein, a method for reconstructing *in-situ* the 3D refractive index from color Schlieren images was also proposed [43].

Additional tricks were also developed to make VAM more practical. One is about the lensing distortion from the cylindrical vial. Usually, either an index-matching bath around the print volume or a cylindrical lens mitigates this lensing effect. However, this can be taken into account when computing the illumination patterns as demonstrated in [44] by resampling the parallel-beam radon transform into an aberrated geometry using some ray tracing analysis. It makes the printer more flexible and easier to use.

The surface quality of the final print is also very important. Tomographic VAM should be better than layer-by-layer technologies regarding that but it suffers from striations, similar in appearance to tens of microns thick layers. It was shown in [45] that these striations are caused by a self-written waveguide effect, driven by the gelation material nonlinearity. The authors proposed a simple and effective method of mitigating striations via a uniform optical exposure added to the end of any VAM printing process.

**Figure 2:**
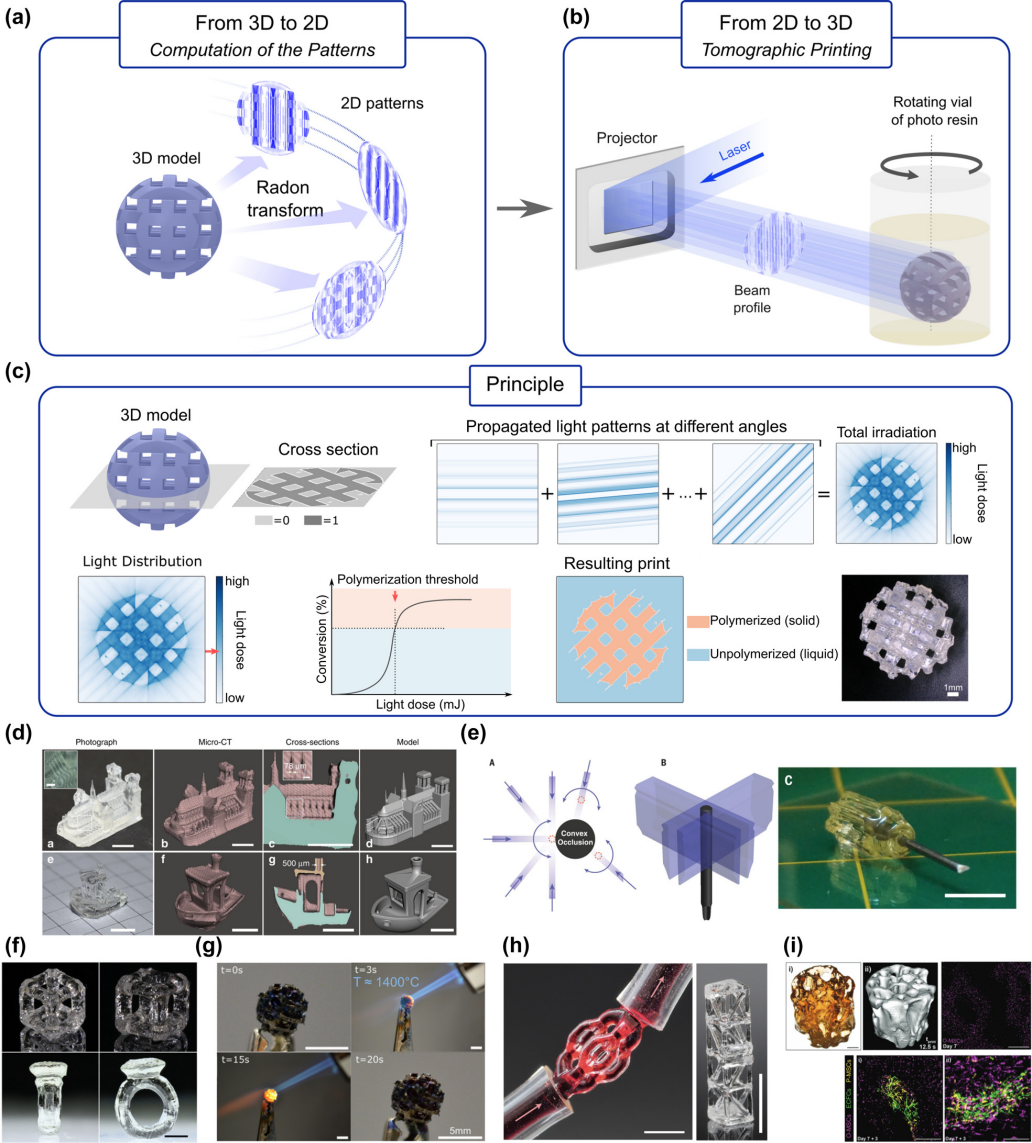
Volumetric additive manufacturing as tomographic back-projection. (a) Radon transform allows calculating the set of 2D tomographic patterns from the 3D model. (b) The back-projection of these patterns into a rotating vial containing a photosensitive resin triggers its solidification. (c) **Tomographic VAM** exploits the nonlinear thresholded response of corresponding photosensitive materials to light-induced polymerization. This polymerization threshold ensures the fabrication of the target object only, even if the resin outside the object’s target volume inevitably receives some light after having been illuminated from multiple angles. The liquid unpolymerized resin can be washed away after the print. Tomographic VAM has been used to (d) produce high-resolution support-free structures (taken from [36], Copyright Springer-Nature); (e) overprint around pre-existing solid objects (taken from [37], Copyright AAAS); fabricate (f) objects with tunable mechanical properties from thiol-ene resins (taken from [48], Copyright Wiley), (g) heat-resistant polymer-derived silicon oxycarbide ceramics (rearranged from [50], temperature indicated, Copyright Wiley), (h) nanoparticle-based silica glass devices (taken from [51], Copyright AAAS); and (i) bioprint cell-laden hydrogels (taken from [46], Copyright Wiley). Scale bars: (d, f, g) 5 mm, (e) 10 mm, (h) 2 mm, (i) (from top left to bottom right) 2 mm, 1 mm, 500 μm, 250 μm.

Volumetric tomographic additive manufacturing has been used in the support-free fabrication of complex-geometry devices. First demonstrated in acrylates and elastomers, tomographic VAM enables the fabrication of functional objects, such as a fluidic ball-cage valve with free-floating elements [46] or overprinting of 3D geometries around preexisting solid components [37], with resolutions below 80 μm [36]. Acrylate chemistry exhibits a nonlinear/thresholded response to light dose, as seen in Figure 3.1.c, thanks to oxygen inhibition [47], and thus is well-adapted for tomographic VAM. Thiol-ene chemistries, in which resins exhibit lower refractive index changes upon polymerization, can be mediated with an inhibitor, such as TEMPO (2,2,6,6-Tetramethylpiperidin-1-oxyl) to exhibit a thresholded behavior. Thiol-ene polymerization has been used to fabricate pieces with tunable mechanical properties, as seen in Figure 2(f) [48], and rapidly cross-linking cell-compatible gelatin-norbornene hydrogels [49].

The technology has also been used to fabricate heat-resistant silicon oxycarbide ceramic devices, as in Figure 2(g) [50]. Here, a siloxane was mixed with an acrylate crosslinker to produce a photosensitive resin that could then thermally transform into a ceramic in a furnace. Additionally, Toombs and coworkers have demonstrated that a polymerizable acrylic backbone can be loaded with silicon dioxide nanoparticles to produce silica glass devices with roughness down to 6 nm (Figure 2(h)) [51].

Tomographic VAM has also been used to cross-link cell-compatible methacrylated hydrogels to produce cell-laden trabecular bone structures shown in Figure 2(i) [46]; bone heterocellular structures replicating vascularization [52]; and organoid-laden, gel-based, biofactories capable of liver-specific ammonia detoxification [40].

## 4 3D optical memories

Another well-known way for going back from 2D to 3D is through 3D optical memories. Here, the goal is to define a 3D distribution to store many 2D data pages or mappings by modifying the optical properties of the media. Unlike volumetric printing, here the idea is to satisfy a 2D-2D mapping rather than the geometric fidelity. Establishing particular 2D-2D mappings is also the goal of photonic circuits or networks, as we will investigate more in the next section. Moreover, it is conceivable to expect an optical memory for fast computation rather than having the memory in electronics. Before moving on to that, we propose to first revisit the “classical” techniques to obtain 3D optical memories.

**Figure 3:**
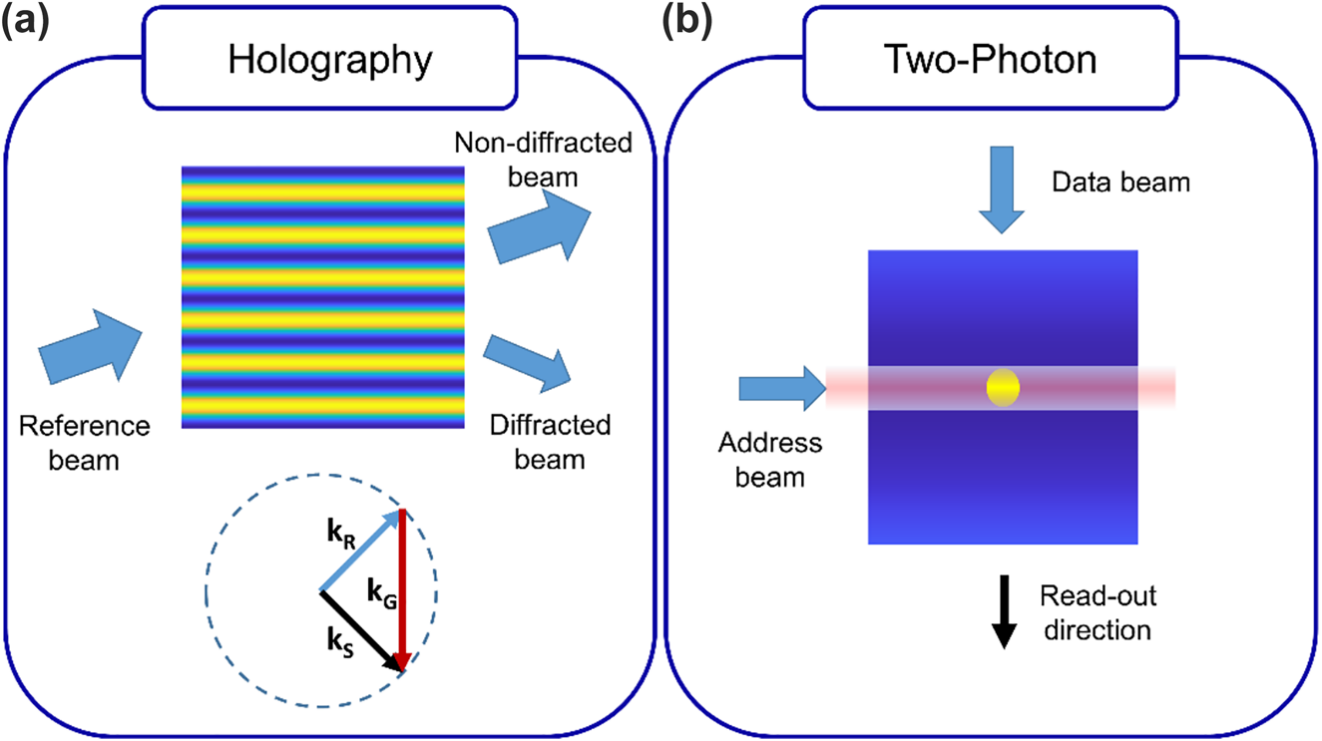
3D optical memory implementations. (a) Diffraction from a sinusoidal grating according to Bragg matching condition. On Ewald’s sphere representation, *k*_R_, *k*_S_, and *k*_G_ refer to the wave vectors of the reference, signal, and recorded grating respectively. The reference beam simply addresses and reads out the data stored in the grating. (b) Simple sketch of recording and read-out for a two-photon technique. Here, the address beam (analog of the reference beam in the case of holography) is depicted as a light sheet accessing a layer of the volume and the data beam encodes the information. During the read-out, the address beam selects the target layer to excite a fluorescence signal that would be modulated with respect to the recorded data (following the description in [57]).

The motivation behind the benefit of using 3D volumes to store 2D data is quite intuitive: the extra degree of freedom provided by the third dimension entails an increase in the storing capacity as compared to 2D layouts. On the other hand, one has to simultaneously deal with cross-talk limitations emerging in tomography and additive manufacturing, i.e. one must be able to access and record data in an isolated way. We can separate 3D optical memories into two main groups concerning the way the data recording and read-out are handled to address this issue: holographic access techniques and two-photon access techniques [53]. In the holographic methods, one piece (analog or discrete) of data is distributed throughout the whole volume whereas one bit of data is stored in a localized spot in two-photon methods. For the sake of completeness, it is worth mentioning that there are other proposals such as persistent spectral hole burning yielding a response in temporal frequency domain [54, 55], but we will limit ourselves to the spatial domain approaches hereafter. Although we referred to holographic data storage as a distributed way of storing data since gratings are recorded in the whole encoding volume, it must be noticed that this process just consists of localizing the data in the spatial frequency domain instead of real space. In Figure 3(a), we show this phenomenon by using Ewald’s sphere representation. Ewald’s sphere is a conceptual construction of a sphere whose radius is equal to the momentum of light. When we place the *k*-vector (momentum) of incident light between the center and the surface of the sphere, the grating vector must connect the tip of the incident *k*-vector onto the sphere to satisfy the conservation of momentum. In Figure 3(a), we show the grating vector as a well-defined (or localized) vector, which would satisfy the Bragg condition only for a specific angle with a given wavelength. The amplitude of the sinusoidal grating would store an analog value, which can be read out with the reference beam as shown in Figure 3(a). The conventional way to obtain such gratings is by optical interference of two plane waves. The obtained hologram is thus transferred to the photosensitive recording material as the 3D variation of the intensity generates a similar variation in some optical properties such as absorption or refractive index [56].

In the case of a two-photon technique, the interaction volume is localized using two-photon absorption that scales with the square of the intensity. On top of that, crossing two orthogonal beams, as shown in Figure 3(b), to satisfy the required intensity to initiate two-photon absorption further narrows the focus volume in comparison with a single beam that has an ellipsoidal point spread function elongated in the optical axis [57, 58]. The local modification obtained by two-photon absorption serves as a written bit of data. During the read-out, the address beam, which could be a light sheet, excites a specific page in the volume and the fluorescence signal modulated with respect to the recorded data is subsequently detected. Selecting a specific volume for recording and read-out provides parallel access and prevents inter-layer interference of different data pages at the same time. We also note that optically induced dielectric breakdown of glass could serve as a localized way of recording and reading data in 3D [59].

**Figure 4:**
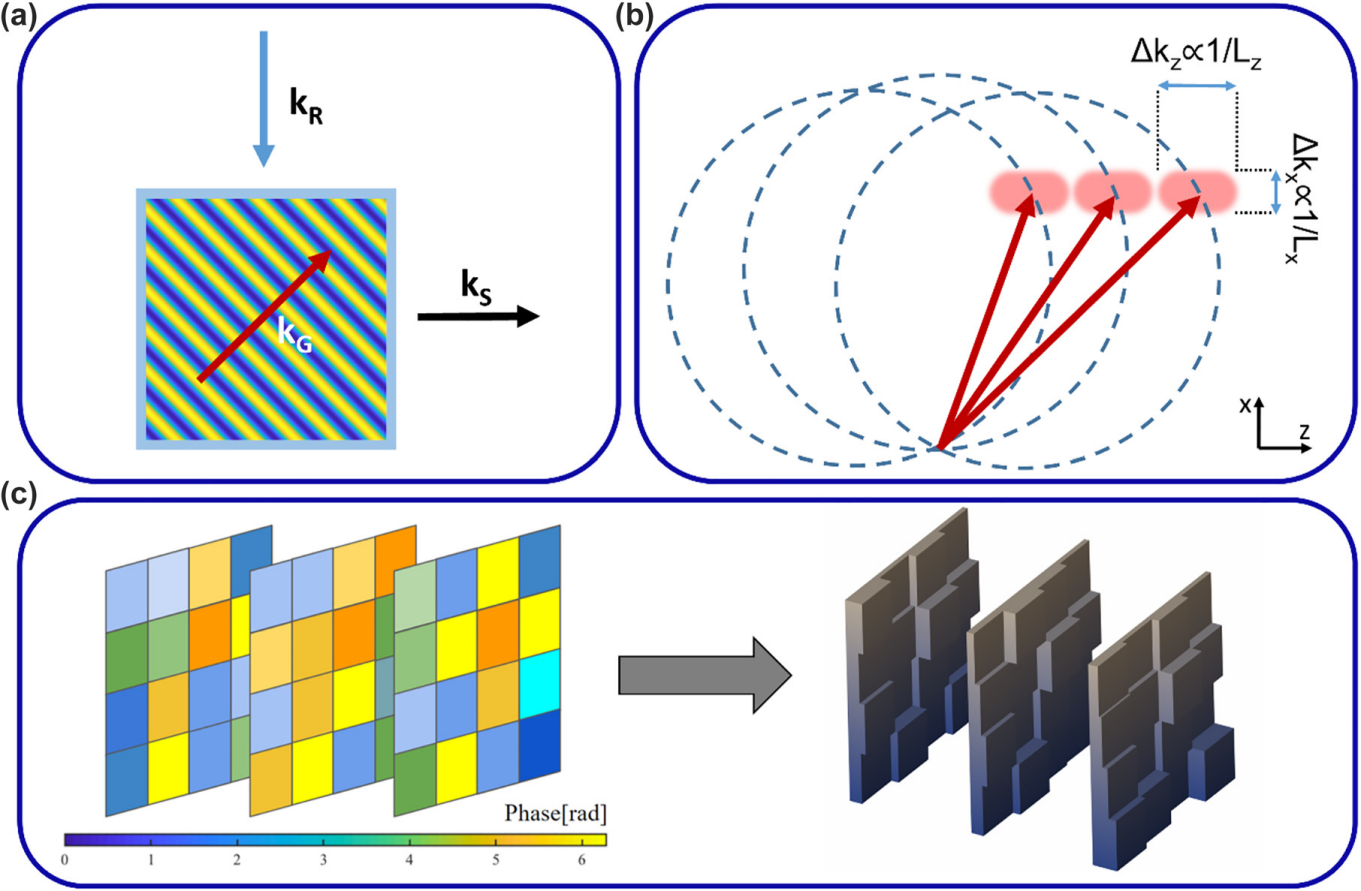
Different holographic strategies. (a) 90° geometry decoupling the non-diffracted beam and modulated diffracted beam. *k*_R_, *k*_S_, and *k*_G_ refer to the wave vectors of the reference, signal, and recorded grating, respectively. (b) Bragg-selectivity in *k*-space separates the different pages of data by mapping them on different Ewald’s spheres due to the different carrier frequencies. The vector clouds are designated by the shaded regions where the size of the cloud inversely depends on the dimensions of the volume hologram, *L*_*x*_ and *L*_*z*_, as shown. The same argument applies to the *y*-direction as well. (c) Schematic for a phase mask stack. The stacked phase masks exhibit volumetric properties when the separation between them is large enough for Fresnel propagation to take place. The varying phase can be encoded as varying thicknesses, which enables the fabrication with a binary-index approach.

To record many data pages in the two-photon system, one should consider the two-photon absorption cross-section and the intensities of the address and data beams to decide on the distance of adjacent spots of data. One should also consider dynamic focus optics synchronized with the address beam to increase the signal-to-noise ratio in the read-out as the emitted photons would undergo some scattering in the media. For holographic access, we should understand how Bragg selectivity works. When many gratings are superimposed, based on the incidence angle, only the Bragg-matched grating would yield strong diffraction towards a designated area whereas all the rest of the refractive index modulation would scatter the light mainly in the direction of the nondiffracted beam. For instance, having the reference and data beams orthogonal to each other, would yield clean read-outs as shown in Figure 4(a). An infinitely large grating would have a well-defined (or ideally localized) grating vector. However, a finite volume grating would have a so-called grating cloud, which is simply due to the convolution of its Fourier transform by a 3D sinc function because of bounded volume [60]. To record many gratings for multiple pages of data, grating clouds should be well separated to prevent cross-talk as depicted in Figure 4(b). The bandwidth of the data in the recorded page would broaden the recording along Ewald’s sphere. This can be understood simply by considering the angular spectrum, meaning that all the individual spatial frequencies in the data page would launch a plane wave with different angles, which would record different gratings with the corresponding reference beam mapped onto Ewald’s sphere. Changing the angle (polar in spherical coordinate) of the reference beam generates another Ewald sphere with the same radius but shifted (as shown in Figure 4(b)), thus separating the data pages thanks to Bragg selectivity. This method is called angular multiplexing [61]. If we change the wavelength, then of course the radius of Ewald’s sphere will change, yielding wavelength multiplexing. If the data page could form a 4π distribution, then the Bragg method would fill all the *k*-space. However, having a data page forming a 4π distribution is practically impossible. Hence, one can change the azimuthal angle of the reference beam such that the new position is farther apart by the bandwidth of the recorded pages to fill the *k*-space. In this case, the data pages multiplexed along the azimuthal direction would all be Bragg-matched (imagine having a fixed Ewald’s sphere in Figure 4(b) and rotating it along the *z*-axis) but they will simply form the reconstructions along different directions. Having a fixed detector with the numerical aperture matched with the bandwidth of the signals would prevent cross-talk. This approach is called peristrophic multiplexing [62].

When we fill the *k*-space with different gratings that are modulated by data envelopes, we can also display a different pattern rather than a reference beam to access the recorded data. Depending on the spatial and angular distribution of the displayed pattern, the superimposed modulated gratings would diffract some portion of the incoming beam, which turns the volume hologram into a correlator with respect to the recorded data in it [63–65]. Moreover, the recording phase of the volume hologram could be arranged in a way that the volume hologram satisfies independent linear connections between the input and output plane, which would resemble the linear weights of neural network architectures [66]. Hence, a volume hologram becomes a natural candidate for a part of photonic circuitry. One bottleneck is the efficiency of individual reconstructions as they decrease with respect to the square of the number of recorded data pages with the explained conventional way of recording [67]. In [68], recording localized holograms in doubly doped LiNbO_3_:Fe,Mn demonstrated a linear efficiency relation. This method can be seen as a hybrid way of recording a 3D memory using holographic and two-photon access at the same time enabling also selective erasure [69].

Another approach to increase efficiency is multilayered systems such as multilayer of phase masks. A phase mask is a 2D variance of phase delay, which gives a shift-invariant response with respect to the excitation angle. By stacking multiple planes, one can destroy shift invariance and introduce multiplexing schemes. With recent advances in additive manufacturing such as two-photon polymerization [70, 71], it has become possible by expressing the phase masks in terms of topography variation, as exemplified in Figure 4(c), and fabricating the stack [72]. As we will delve into the details in the next section, the calculation for such a stack does not have a direct solution and requires iterative methods since the relation of phase modulation with the output field is nonlinear even though the 3D structure provides a linear transform between input and output fields.

## 5 Recent approaches using neural network learning

**Figure 5:**
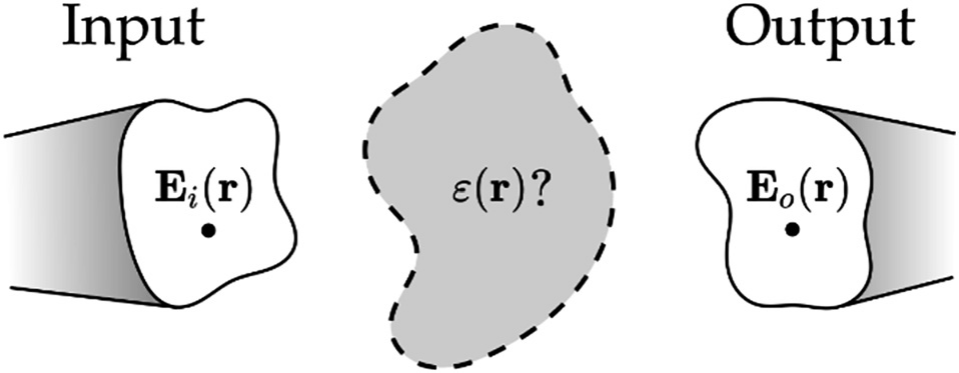
Optical interconnections design. The goal is the determination of geometrical and material properties of the central grey volume that maps input $E_{\text{i}}\left(r\right)$ to output $E_{\text{o}}\left(r\right)$ electric fields with maximal efficiency and minimal cross talk.

In Section 2 we discussed how, given an unknown 3D object, it is possible to extract its geometrical and electromagnetic properties by collecting several 2D projections under different excitation conditions. The methods developed with this diagnostic approach, in which the object under study is fixed and we have free control over the excitation source and collection channel, pave the way to the design of photonic devices where the question is reversed: given a fixed source or a set of input channels, how should I shape matter and choose its electromagnetic properties to obtain the desired output? The answer to this question is at the very essence of many devices such as optical interconnections, multiplexers, couplers, optical filters, spatial and time modulators, optical computers, and so on. We review in the following the main approaches adopted for the design problem sketched in Figure 5: we assume to have one or multiple input channels described by the input electric field distribution $E_{i}\left(r\right)$ on a 2D plane, and we have to determine the electric permittivity distribution *ɛ*(**r**) that gives the target output $E_{\text{o}}\left(r\right)$ at another 2D plane.

For instance, the input may be associated with the modes of an incoming fiber, which should be mapped to or combined with the modes of another fiber. Realizing such an optical interconnect represents an archetypal problem since exploiting free propagating light instead of electrical wiring would result in lower energy consumption, faster communication, and larger parallelizability. The analysis presented in this section holds not only for data transfer and processing but also for optical memories presented in the previous section. In addition, we restrict the problem here to electric fields and currents, but one can straightforwardly extend it to magnetic materials.

The goal of the design problem shown in Figure 5 is to provide the maximal coupling efficiency between a large number of input and output channels within the smallest volume. To make a comparison with biology, the optical interconnect plays the same role as a synapse in a neuron [66]. In this sense, the 3D structure of optical volume elements (OVEs) is promising to overcome electronic implementations as the added degrees of freedom enable maximization of the number of optical modes that can be multiplexed [73, 74]. Here, we stress the term OVE to make it clear that the mentioned optical element has transmission and reflection properties that strongly depend on the spatial and spectral shape of the input field because of the volumetric nature of the optical element.

**Figure 6:**
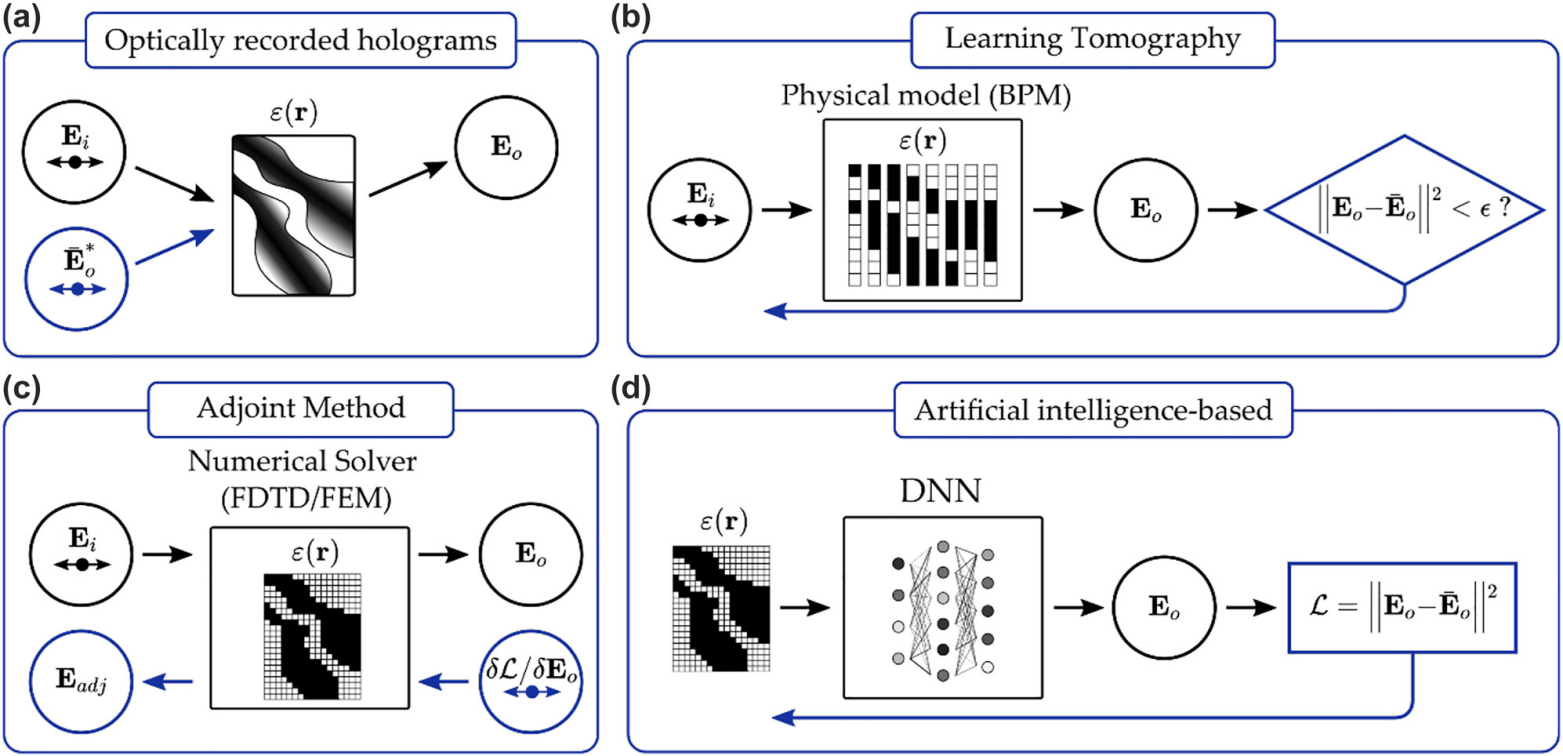
Different approaches for inverse design of volume optical elements. (a) Optically recorded holograms obtained from the interference of incident field **E**_i_ (black) and the conjugated objective field ${\bar{\text{E}}}_{\text{o}}^{*}$ (blue). (b) Learning tomography. The input field is propagated through the guess structure by BPM (black). The predicted output **E**_o_ is compared with the target field ${\bar{\text{E}}}_{\text{o}}^{*}$ and the error is backpropagated to iteratively update the structure (blue). (c) Adjoint variable method: the gradients of the objective function with respect to design parameters are computed through two simulations. The forward one (black) and the adjoint in which the source depends on the original fields and objective function and the corresponding time-reversed simulation (blue). (d) AI-based methods: a DNN maps the relationship between permittivity and output fields (black). The loss is computed as in (b) and backpropagated through the network (blue).

The first fabrication option, as we investigated in the previous section, is to optically record the volume hologram given by the interference of the input field **E**_i_ and the complex conjugate of the objective field at the output **E**_o_, see Figure 6. The technique is usually implemented with photosensitive polymers or photorefractive crystals [75] and the number of exposures required to couple *N* input with *N* output channels is of the order of *N*^2^. The total number of recorded hologram scales as *V*/*λ*^3^ where *V* is the crystal volume and *λ* the recording wavelength. While increasing *N,* the cross-talk among different channels due to undesired diffraction orders represents the main bottleneck of the method [76].

Optically recorded devices can be outperformed by computer-generated holograms (CGHs) in terms of efficiency. Iterative approaches developed for 2D CGHs can be extended to volume holograms. In Ref. [77] the authors propose a method similar to the Gerchberg–Saxton algorithm where, instead of iteratively going from 2D near to a 2D far field, they go from the 3D direct scattering potential to the inverse space and fill the Ewald’s sphere under Born approximation. As introduced in the previous section, multiple phase masks can be stacked to have multiplexing or a correlator that can separate different features as an alternative to continuous volumetric approaches. Another way of thinking about this is distributing the memory in multiple planes, where the diffraction between the planes yields volumetric optical properties as demonstrated using spatial light modulators (SLMs) where the layers are optimized by a general version of the Gerchberg–Saxton iterative optimization algorithm [78].

**Figure 7:**
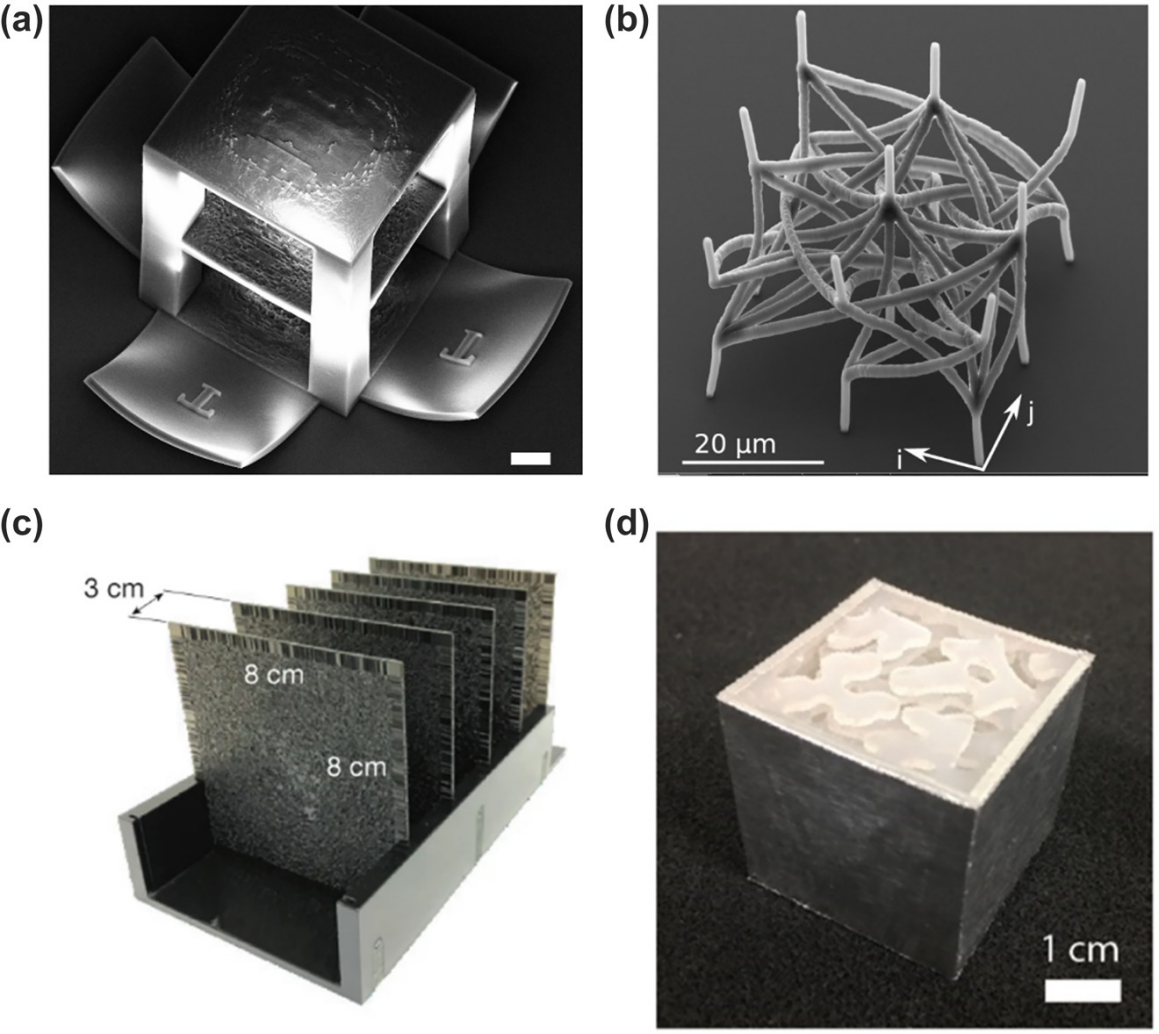
Different modalities for 3D optical circuitry. (a) Multilayer computer-generated optical volume element as an interconnect working in the optical domain printed by two-photon polymerization. The scale bar measures 20 μm (Taken from [72], Copyright De Gruyter). (b) Waveguide interconnects with complex 3D routing to perform image-processing filters (Taken from [80], Copyright Optica). (c) Diffractive deep neural network for various classification tasks experimentally demonstrated in the THz regime (Taken from [81], Copyright AAAS). (d) Volumetric element optimized by adjoint method for wavelength and polarization sorting experimentally demonstrated in the THz regime (Taken from [90], Copyright Optica).

The analogy between ODT and OVE design is intriguing as one can imagine this latter process as the 3D reconstruction of an unknown object of which we know just the 2D projections (the desired output fields **E**_o_) for given incident conditions (the known input fields **E**_i_). Similar to ODT, the efficiency of iterative algorithms strongly depends on the physical model used to simulate wave propagation. As discussed in Section 2, whenever the refractive index contrast is low and Fresnel reflections are negligible, the split-step beam propagation method (BPM) represents a convenient computational tool. Learning tomography was demonstrated as a design algorithm to be combined with additive manufacturing [72] so that the multilayer approach is realized without active devices such as SLMs. The OVE is discretized as a stratified medium where every voxel in each layer provides a phase delay proportional to its refractive index. The output field **E**_o_ computed with BPM is compared with the target ${\bar{\text{E}}}_{\text{o}}$ for all excitation conditions and the error is backpropagated to update the value of the refractive index in each voxel. In this case, unlike from ODT where any prior knowledge on the sample is added through a regularizer term, the designed element is updated at each iteration according to the fabrication constraints. Two-photon polymerization is used in a conventional binary way (either polymerized or not polymerized) that yields a binary index structure, forcing the design into a multilayer element as shown in Figure 7(a). Moreover, having BPM as the forward model enables us to directly optimize the topography rather than the 2D phase masks. Hence, multiple scattering is also captured during the optimization, which further increases the fidelity. In this framework, additive manufacturing through two-photon polymerization was proven as a critical technological step forward, which is compliant with in-plane subwavelength resolutions. By using this platform 3D waveguide interconnects have been experimentally demonstrated [79, 80] (see Figure 7(b)).

Being able to backpropagate light using the time-reversal scheme in Fresnel formulation yields a striking resemblance with the error backpropagation algorithms used in neural networks [24]. Lin et al. [81] introduced diffractive deep neural networks (see Figure 7(c)) by using many examples from a large dataset and back-propagating the error using machine learning algorithms and Fresnel propagation as the forward model. The linear transform performed by multiple layers combined with the absolute square nonlinearity of the detector produced very competitive accuracy results. This method is also applied to different areas from pulse shaping [82] to computational imaging [83]. Following a similar approach, Zhou et al. [84] demonstrated the diffractive processing unit that consists of a digital micromirror device (DMD), an SLM, and a detector. In the unit, data is injected via DMD and bias terms are introduced via SLM, where the free space propagation relays the modulated field to the detector that reads the intensity. Cascading this unit by feeding the detected signal back into DMD, the authors demonstrated a recurrent implementation to perform human action recognition. Other interesting applications where the optical implementations solve algorithmic problems include phase recovery [85] and increasing the resolution of displays [86] using diffractive layers.

For complex structures for which BPM fails, more accurate numerical models, such as finite differences and finite elements, are needed. However, the nonlinear numerical solvers rely on matrix inversions that are not differentiable. In turn, the calculation of objective function gradients with respect to the design parameters is not straightforward as for BPM and it would require a numerical simulation for each derivative of the objective function with respect to a single parameter, e.g. the refractive index value in a voxel. The workaround for topology optimization is represented by the adjoint method [87, 88]. By exploiting Lorentz reciprocity, the gradient with respect to all the input variables can be computed through two successive simulations: a forward one, and an adjoint in which the source term is proportional to the gradient of the objective function with respect to the original fields. Once the derivatives have been computed locally, gradient descent is adopted as in LT for the search of local minima/maxima. The method, largely applied in the nanophotonics community [89], was recently implemented for the optimization of wavelength and polarization splitting OVEs [90] (see Figure 7(d)). The most delicate operation of this approach is the derivation of the adjoint variable formalism. It was recently demonstrated that this step can be also combined or replaced by the same auto-differentiation algorithms developed in machine learning [91].

The similarity of the adjoint variable method and LT scheme with deep neural networks brings us directly to the fourth option for the inverse design of OVE shown in Figure 6. The highly nonlinear relationship between dielectric constant and electric field can be mapped with a digital neural network. In the early stages this was done by collecting a large amount of input-output pairs through numerical simulations, and successively training the network through a direct data-driven approach [92]. Deep enough networks trained with a massive dataset can in this case replace physics-based optimizations for the fast computation of gradients through backpropagation. Recently, different approaches have been proposed to overcome the burden of data collection. Lim et al. [31] proposed to replace the data-driven loss with a physics-based metric by numerically evaluating the residual of Maxwell’s equations on the predicted field from the network. Such indirect training allows for avoiding numerical simulations. Importantly, it also provides a quantitative evaluation of the capability of the network in returning fields that satisfy Maxwell’s equations, instead of just creating an interpolation between input and output images. Although the training remains the most time-expensive process and it requires scanning a large space of parameters before the network is able to generalize to unseen distributions, inference time and gradient computation are an order of magnitude faster than the adjoint method or LT.

As an alternative, deep neural networks (DNNs) have been proposed for the solution of partial differential equations [93, 94]. In this case, the input is not the permittivity distribution but independent variables, such as time and spatial coordinates, and backpropagation is used to rapidly compute the derivatives of the output fields with respect to these latter ones and construct a physics-based loss. Chen et al. [95] demonstrated such physics-informed neural networks for the inverse design of cloaking metamaterials. In contrast with the previous implementation, the network is trained for satisfying Maxwell’s equations and minimizing the difference between output and target fields for a single permittivity distribution and the training has to be performed from scratch for every design task. In both cases, the ability of DNN in mapping deeply nonlinear functions in high dimensional spaces embodies a key ingredient for the realization of 3D optical devices with complex functionalities. Another key concept that makes employing neural networks in the design process is the ability to express high dimensional computational volumes (one can assume the number of voxels as the number of dimensions in the optimization problem) in smaller dimensions, or in other words in latent space representation [31, 96]. This paves the way to optimize large objects that require a heavy computational cost for even a single-pass simulation with the finite difference or finite element methods.

## 6 Conclusion and outlook

Neural networks are emerging as an effective tool for the design of photonic circuits. Tomography, on the other hand, has a longer history and tackled some of the problems already. Therefore, photonic circuit design has a lot to learn from tomography. Another interesting relation arises with tomography when we consider the transmission matrix approach [97]. One can probe the response of a 3D medium by using different inputs (illuminations) and construct the transmission matrix mapping input to output patterns, enabling to structure illumination for a desired response [98]. Once the transmission matrix is obtained, one can use tomography algorithms to figure out the 3D distribution since the required projections can be extracted from the matrix. This equals to say that one can design a transmission matrix providing the desired mapping and use tomography tools to obtain the 3D media. This clarifies the strong connection of tomography with the 3D photonic circuit design.

On the other hand, these circuits can be used to alleviate the heavy digital computations. It was recently demonstrated, for instance, phase recovery [85] by optically implemented networks, which can provide some portion of the required information for optical tomography. Phase recovery, unwrapping, and combining different streams of data from different projections yield a computational burden, which is quite heavy considering that the given problem is, in addition, ill-posed and nonlinear. Optical networks can accelerate the computation by pre-processing the data, which would not require an additional electrical-to-optical conversion as the data is already in the optical domain.

The design of 3D circuits can be often cumbersome and subjected to strong technological constrains. Here, additive manufacturing techniques come to the rescue for the fabrication of complex 3D shapes. Considering the resolution, two-photon polymerization appears to be the needed tool since features comparable to optical wavelengths can be printed. Moreover, graded-index optical elements are also demonstrated using two-photon polymerization [99, 100], which increases the degrees of freedom by introducing the refractive index variance on top of the geometrical degrees of freedom. However, the point-scanning nature of two-photon polymerization yields long fabrication times, making commercial-grade manufacturing challenging. To speed up the fabrication combining one-photon and two-photon techniques is also proposed [101]. From the fabrication time aspect, volumetric additive manufacturing lays a very promising route. The Radon transform-based inverse tomographic approach already provided sub 100 µm resolutions, which is striking considering the centimeter scale of the printed objects. Employing tomographic algorithms that incorporate the effects of diffraction might further increase the resolution while maintaining the fast fabrication scheme of volumetric printing, making it a future-candidate fast approach for the fabrication of photonic circuits.

Lastly, we reviewed the recent studies on 3D optics for functional mappings considering the various design approaches and algorithms, namely starting from optical interference for 3D optical memories to adjoint optimization, learning tomography, data-driven error backpropagation through a physical forward model, and physics-inspired deep neural network implementation.

Considering the computational difficulty of the classical numerical tools, neural networks are becoming an attractive tool for the 3D optics and photonic circuit design as they have already become for tomography to solve the fundamental challenges of 3D-2D transformations. The fact that improvements in the photonic circuitry would yield accelerated and power-efficient neural network architectures tends to remove the boundary between these two disciplines.
